# Role of Peripheral and Central Insulin Resistance in Neuropsychiatric Disorders

**DOI:** 10.3390/jcm13216607

**Published:** 2024-11-03

**Authors:** Kannayiram Alagiakrishnan, Tyler Halverson

**Affiliations:** 1 Department of Medicine, University of Alberta, Edmonton, AB T6G 2R3, Canada; 2 Department of Psychiatry, University of Toronto, Toronto, ON M5T 1R8, Canada; halverso@ualberta.ca

**Keywords:** brain insulin resistance, dementia, depression, exercise, antidiabetic drugs

## Abstract

Insulin acts on different organs, including the brain, which helps it regulate energy metabolism. Insulin signaling plays an important role in the function of different cell types. In this review, we have summarized the key roles of insulin and insulin receptors in healthy brains and in different brain disorders. Insulin signaling, as well as insulin resistance (IR), is a major contributor in the regulation of mood, behavior, and cognition. Recent evidence showed that both peripheral and central insulin resistance play a role in the pathophysiology, clinical presentation, and management of neuropsychiatric disorders like Cognitive Impairment/Dementia, Depression, and Schizophrenia. Many human studies point out Insulin Resistance/Metabolic Syndrome can increase the risk of dementia especially Alzheimer’s dementia (AD). IR has been shown to play a role in AD development but also in its progression. This review article discusses the pathophysiological pathways and mechanisms of insulin resistance in major neuropsychiatric disorders. The extent of insulin resistance can be quantified using IR biomarkers like insulin levels, HOMA-IR index, and Triglyceride glucose–body mass index (TyG–BMI) levels. IR has been shown to precede neurodegeneration. Human trials showed current treatment with certain antidiabetic drugs, as well as life style management, like weight loss and exercise for IR, have shown promise in the management of cognitive/neuropsychiatric disorders. This may pave the pathway to the development of new therapeutic approaches to these challenging disorders of dementia and psychiatric diseases. Recent clinical trials are showing some encouraging evidence for these pharmacological and nonpharmacological approaches for IR in psychiatric and cognitive disorders, even though more research is needed to apply this evidence into clinical practice. Early identification and management of IR may help as a strategy to potentially alter neuropsychiatric disorders onset as well as its progression

## 1. Introduction

The brain is one of the most metabolically active organs in the human body. Metabolism of glucose is used for synaptic transmission, and any alterations to its metabolism may play a role in neurodegenerative disorders by affecting the mitochondrial activities and ATP production, which can also affect the synaptic connections [1,2]. It has been shown that insulin has significant effects on the brain, particularly where it plays a role in maintaining glucose, fat, and energy metabolism. The brain has insulin receptors through which insulin of peripheral origin or insulin produced in the brain acts on the whole brain. Insulin in the brain contributes to neurotrophic, neuromodulatory, and neuroprotective effects [3,4]. Insulin effects are produced after it binds to its receptors and causes auto phosphorylation of its receptor by activating tyrosine kinases and insulin-like growth factor 1 (IGF-1). It can also initiate neuro trophic actions [5]. Insulin receptors are seen in different areas of the brain such as the olfactory bulb, hippocampus, midbrain, hypothalamus, and cortical areas [6] Insulin activates the insulin receptor (IR), which is a type of tyrosine kinase receptor, in which the binding of an agonistic ligand leads to autophosphorylation of the tyrosine residues. Tyrosine-phosphorylated IR then binds with numerous signaling partners [7], as shown in Table 1. Decreased transport of insulin from peripheral circulation to the brain, and insulin resistance are the result of hypothalamic inflammation due to inflammaging [8].

Different mechanisms, like oxidative stress, mitochondrial dysfunction, and inflammation, play roles in insulin resistance, but the exact mechanisms are still not recognized [10,11]. Not only through multiple mechanisms, but also through different cell types and neural circuits, insulin is able to regulate key brain functions. Insulin is able to regulate the cellular functions of various brain cells, including neurons, astrocytes, microglia, and tanycytes. It can also be involved in different brain pathways, such as the mesolimbic dopaminergic and hypothalamic circuits. Insulin is also involved in modulating global brain processes, such as synaptic transmission, and brain metabolism. When there is a dysregulation in insulin signaling, it can lead to certain brain disorders, including depression and Alzheimer’s dementia (AD) [12].

**Figure 1:**
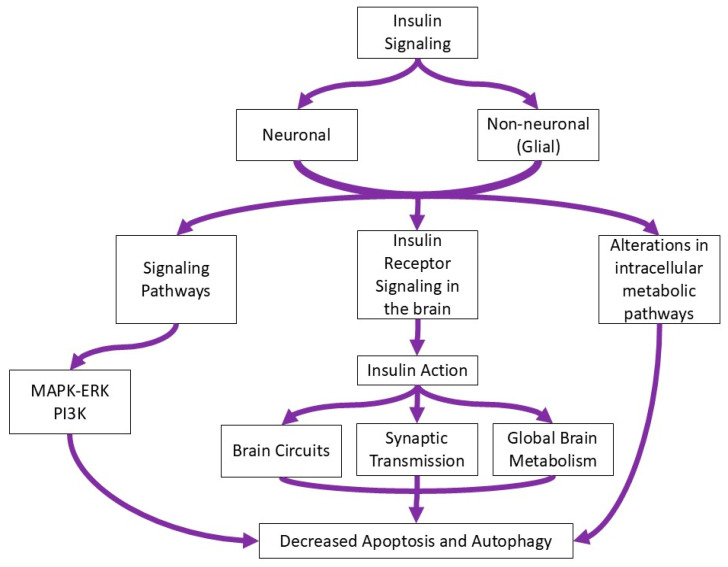
Flow diagram of the role of insulin signaling in the brain.

Insulin receptors are expressed on all cell types (neuron, microglia, astrocytes, oligodendrocytes, BBB) in the brain and plays a role in insulin signaling (Table 1). Brain insulin signaling plays a significant role both in healthy and disordered brains. Insulin signaling occurs through the PI3K-signaling pathway as well as through the MAPK–ERK-signaling pathways, and it contributes to different functions in the brain [13,14]. Investigations about the roles that insulin signaling plays in non-neuronal cell types in the brain showed they can play a role in neuronal metabolism, synaptic plasticity, and neurotransmission (Figure 1) [15,16].

Insulin resistance (IR) is defined as a clinical state in which cells fail to respond normally to the insulin hormone and also denotes impaired insulin sensitivity that impede glucose disposal [17]. Within the cell, insulin resistance occurs at multiple levels from the cell surface to the nucleus. In the nucleus, it can inhibit P13K cascades, resulting in failure of the Foxo1-activate gene transcription profiling [18]. Insulin resistance or metabolic syndrome can be peripheral or central (Brain). In general, peripheral insulin resistance has been associated with hyperglycemia, hypertension, low HDL and increased triglycerides, central (visceral obesity), microalbuminuria, increased fibrinogen, increased plasminogen activator, and increased uric acid levels [19]. With insulin resistance, increased insulin levels (hyperinsulinemia) are seen [20,21]. The prevalence of IR is continually growing and is around 51% throughout the world [22]. With central insulin resistance, the brain cells do not respond, with a decrease in insulin receptors and reduction in the insulin-signaling process [9]. Brain insulin resistance (BIR) is secondary to decreased levels of insulin transport in the blood–brain barrier (BBB) and altered or impaired brain insulin signaling. [23,24]. Hence, BIR is emerging as an important pathophysiological mechanism of diabetes causing cerebrovascular complications, including dementia and neuropsychiatric disorders. In a small study by Abbasi F et al., insulin resistance in otherwise healthy young and middle-aged adults was found to be associated with preclinical signs of neuropsychiatric impairment (cognition and mood) [25].

As there are no good measures to detect BIR, the homeostatic model for insulin resistance (HOMA–IR), which is used to measure peripheral insulin resistance, is still used. Some authors see IR-like features in some cognitively normal subjects and also in asymptomatic APOE ε 4 carriers [26,27,28]. Animal studies also point out that IR can occur with atypical depression [29].

With better understanding of the basic mechanisms of insulin action in the brain, there is the potential in identifying new therapeutic targets for the treatment of various neuropsychiatric conditions. One area of emerging research is through the role of cholesterol synthesis. Acute insulin signaling can regulate de novo cholesterol synthesis in both neurons and glia cells. However, in conditions such as diabetes, where there is hyperinsulinemia and insulin resistance, this can impact the brain insulin sensitivity, resulting in a loss of the normal dynamic regulation of brain cholesterol production. Thus, this may contribute to the acceleration of AD progression, potentially through an altered ApoE metabolism (which has a strong genetic link to the development of AD) [30]. Developing a further understanding of brain insulin and insulin-like growth factor (IGF-1) signaling may help to determine the specific sites that insulin acts on, including the different subtypes of astrocytes and neurons that are involved in the regulation of cholesterol and long-chain fatty acid biosynthesis. This may help to develop a further understanding of how these factors contribute to the development and pathogenesis of brain disorders, such as dementia and depression.

In this review article, we looked at the possible links between metabolic syndrome and changes in mood and cognitive function in the brain mainly through the mechanisms of peripheral and central insulin resistance as well as possible therapeutic approaches to address IR in these conditions.

## 2. Methodology

### Search Strategy

A literature search was performed using the electronic databases MEDLINE (1966–August 2024), EMBASE and SCOPUS (1965–August 2024), and DARE (1966–August 2024). The main search items were cognitive impairment, dementia, depression, anxiety, bipolar disorder, schizophrenia, peripheral insulin resistance, central insulin resistance, symptoms of insulin resistance, and insulin resistance management. This article is a non-systematic, narrative review. Non-English articles were excluded.

## 3. Pathology

Insulin production occurs mainly in pancreatic β-cells, and it regulates systemic metabolism and brain function [31]. Pancreatic insulin can enter the brain through the capillary endothelial cells of the BBB [32,33]. Insulin acts both on neuronal and non-neuronal cells, through the insulin signaling pathways (AKT and MAPK pathways) [34,35]. AKT- and MAPK-insulin-signaling pathways can be activated separately in different medical conditions [36]. Meanwhile, IGF-1 is a hormone, produced by many cell types, and it stimulates tissue remodeling [37,38]. Insulin resistance is primarily caused by the inability of insulin to effectively activate the insulin receptor substrate (IRS). This reduced IRS activation weakens GLUT4 function in the context of glucose transport [10]. This can lead to aberration with oxidative stress, inflammation, mitochondrial dysfunction, and endoplasmic reticulum, which could lead to insulin resistance, but the exact cause is still unknown.

Insulin receptor dysfunction can occur in Alzheimer’s disease. Brain insulin resistance is a core feature of ADRDs [9]. Glucose metabolism alterations lead to a decrease in ATP function, which leads to neuronal and synaptic dysfunction and depletion of citric acid cycle intermediates that are required for the synthesis of acetylcholine in the brain. This may lead to defects in synaptic transmission and proper cognitive function [39,40].

The peripheral IR can lead to central or brain IR possibly due to decreased transport of Insulin across the BBR with a down regulation of the endothelial insulin receptors as well as impaired insulin signaling [41,42]. Different mechanisms like hyperinsulinemia, mitochondrial dysfunction, neuroinflammation, leads to impairment in neuronal function and synaptogenesis. Hyperinsulinemia also increases Aβ deposition as well as neurofibrillary tangles (NFTs) formation, and also have a negative impact on insulin signaling. [9,43].

“Insulin resistance is defined as an impaired response to insulin stimulation in peripheral tissues, which leads to increased peripheral insulin levels” [44]. Brain insulin resistance is defined as the reduced physiological actions of insulin in the brain. In the nerve cells, insulin modulate neurotransmitters like N-methyl-d-aspartate (NMDA) and γ-aminobutyric acid (GABA) as well as catecholamines [45]. There is a bidirectional relationship between peripheral and brain insulin resistance, with each one influencing the other [17,46]. Since the pancreas is the main source of CSF Insulin levels, its level correlate with peripheral levels [47] and altered transport across BBB has been shown in IR [48]. Brain insulin resistance can induce activation of microglia and astrocyte and impair intracellular signaling leading to mood disorders and cognitive impairment [46,49] (Table 2). A decrease in the cerebrovascular cell’s cytoplasmic insulin receptors along with defective coupling are seen in BIR as well as in AD [50,51].

### 3.1. Research Studies Showing Evidence for the Association of IR with Cognitive and Neuropsychiatric Disorders

Selected studies showing association of insulin resistance with cognitive decline/dementia as well as neuropsychiatric disorders are shown in Table 3. In the CAIDE study, which is a Finnish longitudinal study conducted over 7 years in older adults, insulin and insulin resistance (HOMA-IR) were predictors of global cognitive functioning [52]. Different longitudinal studies, like Washington Heights Northern Manhattan Study [53], Honolulu-Asia Aging study [23], and ARIC study (in middle-aged subjects) [54], confirmed these observations. Whereas with the Rotterdam study, the effects of insulin and insulin resistance were seen only in the first three years, when the recruited subjects were followed-up 9.7 years after the study, a possible role in advancing the onset of Alzheimer’s dementia was concluded [55]. In contrast, with the Uppsala Longitudinal Study of Adult Men, serum insulin was not associated with later cognitive decline [56]. In the Hiyasama autopsy study, IR, as measured by fasting insulin and HOMA- IR, was associated with the pathology (neuritic plaques) [57]. TyG index, another marker of IR, showed in the National Health Information Database population study that it was associated with an increased risk of both vascular and Alzheimer’s dementia, even after correcting for the confounding vascular risk factors [58].

Selected studies showing the association of insulin resistance also with depression and other neuropsychiatric disorders are also shown in Table 3. In a cross-sectional study by Pearson et al., insulin resistance, as measured by waist circumference and HOMA-IR, was associated with depression [59]. A study showed that the HOMA IR index was associated with acute depression [60], but the triglyceride–HDL ratio, another biomarker of IR, was shown to be associated with depression severity and chronicity [61]. In the PREVENT cohort study, IR in middle aged individuals was associated with depression and impaired executive function [62]. The Bialystok study showed the association of IR with subclinical depression [63], whereas in a Chinese study on major depressive disorder (MDD), IR was also associated with suicide attempts [64]. Increased insulin resistance is also seen in other psychiatric disorders, such as schizophrenia and bipolar disorder [65,66]. A study showed that treating insulin resistance improves clinical outcome in bipolar disorder [67].

**Table 3:** Selected studies showing the association of insulin resistance with neuropsychiatric conditions.

Study	Participants	Outcome	Reference
CAIDE Study	Individuals aged 65–79-year-old that are dementia-free *n* = 269	Serum glucose and insulin were measured at baseline, with IR estimated with HOMA-IR and re-examined 7 years later with cognitive assessments. Individuals with higher baseline HOMA-IR values were found to have worse performance in global cognition (β [standard error (SE)] −0.050 [0.02]; *p* = 0.043) and psychomotor speed (β [SE] −0.064 [0.03]; *p* = [0.043]) when examined 7 years later. Participants with elevated serum insulin levels had lower scores on global cognition (β [SE] −0.054 [0.03]; *p* = 0.045) and poorer performance in psychomotor speed (β [SE] −0.061 [0.03]; *p* = 0.070).	Hooshmand et al. (2019) [52]
Rotterdam Study	Individuals that are both dementia- and diabetes-free *n* = 3139	Fasting glucose and insulin levels were measured at baseline. HOMA-IR was used to estimated IR. During follow-up, 211 participants developed AD, with 71 individuals developing it within 3 years. Following 3 years, the risk was no longer increased, suggesting that insulin metabolism influences the risk of AD only within 3 years.	Schrijvers et al. (2010) [55]
Washington Heights Northern Manhattan Study	Samples were obtained from people aged 65 years or older (random) *n* = 683 subjects	Individuals were followed for 3691 person–years, with 149 persons developing dementia (*n* = 137: AD, *n* = 6: dementia associated with stroke, and *n*= 6: others with dementia). In the sample population that had hyperinsulinemia (39%), the risk of AD doubled (HR = 2.1; 95% CI: 1.5, 2.9) and was highest in those without diabetes. The HR-related presence of hyperinsulinemia or diabetes in 50% of our sample to AD was 2.2 (95% CI: 1.5, 3.1). The risk of AD attributable to the presence of hyperinsulinemia or diabetes was found to be 39%.	Luchsinger et al. (2004) [53]
Honolulu-Asia Aging Study	Subjects were dementia-free Japanese-American Men (aged 71–91) *n* = 2568	Serum insulin levels were obtained at baseline (year 1991), and they were re-examined in 1994 and 1996. There were 244 new cases of dementia, and they found that the risk of dementia was at the two extremes of the insulin distribution (lower and upper 15th percentiles) (HR = 1.54, CI 1.11 to 2.11 and HR = 1.54, CI 1.05 to 2.26). Men with insulin levels <22.2 mIU/L showed a decreased risk for dementia when there were increased levels of insulin (HR = 0.76, CI 0.72 to 0.79 for each increase in one logarithmic unit −2.72 mIU/L of insulin). In men with insulin levels ≥22.2 mIU/L, the risk for dementia increased in association with increasing insulin levels (HR = 1.64, CI = 1.07 to 2.52 for each 2.72 mIU/L).	Peila et al. (2004) [23]
Prospective Epidemiological Risk Factor Study	Elderly women in Denmark *n* = 2103	It was determined that impaired fasting glucose was associated with a 44% (9–91%) larger probability of cognitive dysfunction. Individuals with a HOMA-IR >2.6 had a 47% (9–99%) larger chance of developing cognitive decline.	Neergaard et al. (2017) [68]
Finnish Nationwide Health Examination Survey	An 11-year follow-up study with adult subjects (mean age 49.3) *n* = 3695	HOMA-IR, fasting insulin and glucose, HbA1c, and hs-CRP were collected at baseline and used as predictors of cognitive performance. Cognitive function was measured using categorical verbal fluency, word-list learning, and word-list delayed recall. Higher baseline HOMA-IR and fasting-insulin levels were independent predictors of poorer verbal fluency performance (*p* = 0.0002) and of a greater decline in verbal fluency during the follow-up time (*p* = 0.004).	Ekblad et al. (2018) [41]
ARIC Study	Middle-aged adults (aged 45–64) without any prior diagnosis of T2DM, stroke, or dementia were assessed between 1987 and 1989. *n* = 7148	Participants were assessed with a fasting insulin and glucose along with three tests of cognitive function (DWR, DSS, and WF) at baseline and 6 years later. Hyperinsulinemia results from fasting insulin and HOMA-IR were associated with significantly lower baseline DWR, DSS, and WF scores and a greater decline over 6 years in DWR and WF.	Young et al. (2006) [54]
Uppsala Longitudinal Study of Adult Men	Swedish adult men (age 71) that were free of dementia between the years of 1990–1995. *n* = 1125	OGTT and euglycemic insulin clamp were determined at baseline. There was a follow-up in 12 years, in which 257 participants developed dementia or cognitive impairment. Low early insulin response to the OGTT was associated with a higher risk of AD (HR for 1 SD decrease 1.32; 95% CI 1.02, 1.69). Low insulin sensitivity was associated with a higher risk of VD (HR for 1 SD decrease 1.55; 95% CI 1.02, 2.35). The authors also found that there was a 63% increased risk of any dementia and cognitive impairment in patients with diabetes.	Rönnemaa et al. (2009) [56]
Hisayama Study	Specimens from 135 autopsies (men = 74, women = 61) between 1988 and 2003.	In total, 75 g OGTT, fasting glucose and insulin, 2 h post-load plasma glucose, and HOMA-IR were measured in 1988. Autopsy samples were assessed for neuritic plaques and neurofibrillary tangles. Higher levels of 2 h post-load plasma glucose, fasting insulin, and HOMA-IR were associated with increased risk for neuritic plaques.	Matsuzaki et al. (2010) [57]
National Health Information Database population study	This was a retrospective, observational cohort study using data from 2009 to 2015 of adults 40 years or older. *n* = 5,586,048	IR was measured with the TyG index, and participants were divided into quartiles based on the results. During follow-up of around 7.21 years, dementia was diagnosed in 142,714 (2.55%) participants. AD and VD were diagnosed in 74.3% and 12.5% of the participants, respectively. Multivariate-adjusted HRs for patients in the TyG index fourth quartile were higher for dementia (HRs = 1.14; 95% confidence interval [CI] 1.12–1.16), AD (HRs = 1.12; 95% CI 1.09–1.14), and VD (HRs = 1.18; 95% CI 1.12–1.23) compared with the first quartile of TyG index.	Hong et al. (2021) [58]
Insulin Resistance and Depression	Participants included adults aged 26–36. *n* = 1732	Fasting glucose and insulin and HOMA-IR were used to measure IR. The 12-month prevalence of depressive disorder was 5.4% among men and 11.7% among women. It was found that the insulin resistance was higher in depressive individuals (17.2% (95% CI 0.7–36.0%, *p* = 0.04) higher in men and 11.4% (1.5–22.0%, *p* = 0.02) higher in women). Overall insulin resistance was associated with a depressive disorder at 13.2% (−3.1 to 32.3%, *p* = 0.12) in men and 6.1% (−4.1 to 17.4%, *p* = 0.25) in women.	Pearson et al. (2010) [59]
Insulin resistance in Depression	A meta-analysis that looked at 70 studies of insulin resistance in depression. *n* = 240,704	The study examined fasting serum and plasma insulin and glucose levels and the HOMA-IR index in people with depression in an acute episode with and without psychiatric medications and in depression during remission, comparing them to the values in healthy controls. Random-effects between-group meta-analyses showed that insulin levels were increased in acute episodes of depression (g = 0.29, 95% CI 0.21–0.37, *p* < 0.001.) Similarly to insulin, the HOMA-IR index was increased during acute depression (g = 0.30, 95% CI 0.18–0.41, *p* < 0.001).	Fernandes et al. (2022) [60]
NESDA Study	Individuals with a history of depression (current MDD, remitted MDD, and no history (control)) *n* = 1269	The QUICKI and the triglyceride to HDL ratio were used to look at the association of IR to MDD. Insulin resistance was associated with current MDD compared with control individuals (odds ratio [OR], 1.51; 95% CI, 1.08–2.12), but not with remitted MDD (OR, 1.14; 95% CI, 0.79–1.64). Triglyceride-HDL ratio was positively associated with depression severity and chronicity, for those with a current MDD diagnosis.	Watson et al. (2021) [61]
Insulin resistance in schizophrenia	Individuals with a first-episode schizophrenia, who were antipsychotic-naive with matched, unaffected controls *n* = 58 patients with schizophrenia *n* = 58 control	The HOMA-IR was used to infer IR, β cell function, and insulin sensitivity from clinical measurements of fasting-glucose and -insulin serum levels. Patients with schizophrenia showed increased baseline HOMA-IR (mean difference [MD] [SE], 0.68 [0.25]; *p* = 0.004).	Tomasik et al. (2019) [65]

Alzheimer dementia—AD, delayed word recall—DWR, digit symbol subtest—DSS, first letter word fluency—WF, hemoglobin A1c—HbA1c, high-density lipoprotein—HDL, high-sensitivity C-reactive protein—hs-CRP, homeostasis model assessment for insulin resistance—HOMA-IR, insulin resistance—IR, major depressive disorder—MDD, oral glucose tolerance test—OGTT, quantitative insulin sensitivity check index—QUICKI, Type 2 diabetes mellitus—T2DM, triglyceride–glucose—TyG, vascular dementia—VD.

### 3.2. Clinical Presentation of Peripheral and Central Insulin Resistance

The clinical presentation of insulin resistance varies with the type as well as the stage of insulin resistance (Table 4). Commonly, they present typical symptoms of diabetes mellitus such as polyuria, polydipsia, polyphagia, weight loss, and the need for large doses of insulin for management. Some present with severe hyperglycemia requiring even >200 units of insulin.

Most patients have one or more clinical features of the insulin-resistant state, as shown in Table 4.

Sometimes, individuals may present different phenotypes of metabolic syndrome. Presentation of this can vary, and, in some patients, despite having some or even most of the components of insulin resistance present, the metabolic syndrome can be asymptomatic. In others, it can present as obesity (most common cause of insulin resistance) or Type 2 diabetes mellitus (chronic or acute), Impaired Glucose Tolerance (IGT)with dyslipidemia, hypertension, macrovascular disease (stroke, coronary artery disease, peripheral vascular disease). A combination of hyperglycemia and virilization occurs with several syndromes of insulin resistance [69,70].

There are two types of metabolic syndrome (MetS). In type A, which affects young women, is characterized by severe hyperinsulinemia, IR, and usually present with obesity, Acanthosis nigricans and features of hyperandrogenism such as hirsutism, acne, frontal baldness and menstrual abnormalities are seen. Some patients present polycystic ovarian syndrome. Type B syndrome (autoantibodies to insulin receptor), where patients present with diabetes mellitus. Agonist activity (hypoglycemia) and antagonist effect (insulin resistance) are both seen in the same patient.

Different criteria are available to diagnose metabolic syndrome: 1. WHO criteria [71]; 2. AACE (The American Association of Clinical Endocrinologists) clinical criteria for insulin resistance syndrome; and 3. IDF (International Diabetes Federation (IDF)) global consensus criteria on metabolic syndrome [72,73].

A diagnosis of metabolic syndrome is conducted after assessing different parameters, like abdominal obesity (waist–hip ratio > 0.9 in men or >0.85 in women; or body mass index (BMI) > 30 kg/m^2^; and lipid profile (Triglycerides 150 mg/dL or greater, and/or high-density lipoprotein (HDL)–cholesterol < 40 mg/dL in men and <50 mg/dL in women.) [74].

IR is commonly seen with certain medical conditions, such as diabetes, non-alcoholic fatty liver disease, polycystic ovarian syndrome, coronary artery disease (CAD), Cerebrovascular Disease (CVD), and Peripheral Arterial Disease (PAD), as well as with diabetic neuropathy, retinopathy, and nephropathy, and with auto-immune diseases like SLE. Assessment for IR should be carried out with these medical conditions, whenever it is necessary [75].

### 3.3. Investigations/Biomarkers for Insulin Resistance and Its Association with Neuropsychiatric Disorders

The hyperinsulinemic–euglycemic clamp technique is the most scientifically sound technique for measuring insulin sensitivity [76]. In practice, measuring for high blood sugars, fasting-insulin levels, and tests of insulin resistance, like HOMA IR (homeostasis model assessment), Triglyceride glucose–body mass index (TyG–BMI), Leptin, Amylin, and Heme oxygenase (HO-1) levels, are also useful. Plasminogen activator inhibitor (PAI)-1, Adiponectin, retinol-binding protein-4 (RBP4), chemerin, and adipocyte fatty-acid-binding protein (A-FABP) have been suggested as potential biomarkers for insulin resistance. HOMA is a common marker used in clinical practice, and IR is defined as a HOMA level > 2.5. [77]. The glucose clamp method is the reference standard for direct measurement of insulin sensitivity. The validated tests, like QUICKI [78,79] and HOMA- IR, are conducted in a steady-state condition (fasting), whereas in dynamic condition (after an oral glucose load), tests like Matsuda Index and Gutt Index [80,81] are used.

In a longitudinal study in elderly individuals without dementia over a period of 7 years, Hooshmand et al. showed that raised serum insulin levels were associated with lower scores on global cognition (β [SE] −0.054 [0.03]; *p* = 0.045 [52]. TyG–BMI is a useful surrogate marker for insulin resistance in nondiabetic individuals and can independently predict new cardiovascular and cerebrovascular adverse events [82,83]. The triglyceride glucose (TyG) index, which is an indicator of insulin resistance (IR), is related to cerebrovascular disease and dementia [84]. A high TyG index was also associated with small vessel disease of the brain in normal individuals [85]. The TyG index has been strongly associated than HOMA IR in predicting IR in certain medical conditions [86] as well as assessing the risk of cognitive decline and its biomarkers [31,87,88].

Among the markers of IR, the TyG index is a cost-effective biomarker of IR [89,90] and has been linked to poorer cognitive function [52,91,92]. Both cross-sectional and longitudinal studies showed the association of IR markers with cognitive decline. In the cross-sectional study by Kong et al., increases in HOMA-IR were related to fasting-insulin levels (*p* = 0.001) and reductions in MMSE (*p* = 0.004) [93], whereas the study by Smith et al. showed that higher HOMA-IR and leptin were associated with lower executive function [94]. The longitudinal study by Fava et al. showed that increased HOMA-IR had lower scores on MMSE (*p* = 0.001) and ADAS-Cog (*p* = 0.001) [95].

A cross-sectional study showed an association of triglyceride glucose index with depression episode [96] as well as suicide attempts [64], and a longitudinal study showed an association with depression progression [97].

### 3.4. Management of Insulin Resistance in Neuro Psychiatric Disorders

New therapeutic approaches to brain disorders centered around (1) insulin signaling, (2) brain metabolism, and (3) overcoming brain insulin resistance are seen. No medications are specifically approved to treat insulin resistance. The general treatment approaches in managing insulin resistance include altering lifestyle factors through (1) diet, (2) intermittent fasting, (3) exercise/physical activity, (4) smoking, (5) alcohol intake, and (6) antidiabetic medications. These interventions may help to enhance insulin sensitivity and also regulate the levels of insulin and glucose in these neuropsychiatric disorders [98,99].

1. Diet:

In animal studies, exercise and a healthy diet have been shown to improve brain insulin sensitivity [100]. Weight loss can help to improve insulin resistance [101].

2. Intermittent Fasting/Caloric Restriction:

Dietary approaches such as intermittent fasting and caloric restriction are linked to both improved insulin sensitivity and neuroprotection, suggesting a beneficial effect on metabolic and neuronal pathways [102,103]. In a recent small randomized clinical trial on cognitively intact insulin resistance older adults, intermittent fasting for over 8 weeks showed improved insulin-signaling biomarkers [104].

3. Exercise:

Many studies have shown that exercise can improve insulin resistance [105,106,107]. A small study showed that an 8-week exercise intervention in sedentary individuals can restore insulin action in the brain [108]. Both animal and human studies point out how exercise can improve brain sensitivity [109]. Exercise may improve IR by regulating different molecular mechanisms such as mitochondrial function and autophagy [110] Among all the strategies to treat insulin resistance, a non-pharmacological approach with exercise is a simple, cost-effective measure to improve insulin sensitivity.

Exercise can increase AMPK activity and GLUT 4 translocation in muscle cells and thereby help to reduce IR [105]. In a meta-analysis study by Fedewa et al., exercise training in youth improves insulin resistance [111]. A systematic review and meta-analyses by Sampath Kumar A et al. provide useful information on how a structured exercise program improves IR [112]. A recent RCT also showed that a structured exercise training program for only 12 weeks can effectively reduce insulin resistance in middle age and older adults [113]. Overall, it seems that exercise may be a practical and reliable intervention in all age groups to overcome IR.

4. Smoking:

Smoking has been linked to insulin resistance, and nicotine is associated with decreased insulin sensitivity. Smoking also increases the risk of insulin resistance in a dose-dependent manner [114,115,116,117]. Smoking cessation or reduction may help to improve insulin sensitivity [118,119].

5. Alcohol intake:

Light-to-moderate alcohol consumption on fasting-insulin and IR are dependent on, but independent of, obesity and depression symptom severity [120].

6. Antidiabetic medications:

Antidiabetic agents like (1) metformin, (2) thiazolidinediones, (3) DPP-4 inhibitors, (4) SGLT inhibitors, and (5) intranasal insulin are shown in clinical studies to be helpful in reducing IR, and this has been discussed in Table 5.

Metformin improves insulin resistance by increasing the recruitment and activity of the glucose transporter GLUT4 as well as improving tyrosine kinase (insulin receptor) activity [121]. An animal study by Ruegsegger et al. showed that exercise and metformin restore brain mitochondrial function in insulin-resistant states [122]. For thiazolidinediones, pioglitazone and rosiglitazone showed some promising effects on IR, more so with pioglitazone, but health care professionals should weigh the risk–benefit with the heart failure and bone fracture side effects seen with these agents [123,124] SGLT2 inhibitors (like canagliflozin, dapagliflozin, and empagliflozin) showed beneficial effects in reducing IR [125]. GLP-1 receptor agonists like (liraglutide, semaglutide, dulaglutide, and exenatide) can improve insulin sensitivity by reducing the inflammatory response of macrophages [126], whereas DPP-4 inhibitors (such as gemigliptin, saxagliptin, sitagliptin, teneligliptin, trelagliptin, vildagliptin) could improve insulin sensitivity by reducing the activation of down-stream AKT signaling [127].

While small, early clinical trials with intranasal insulin showed some cognitive benefits [128] and mood benefits [129], RCT study did not show cognitive benefit [130] or mood benefit [131] with intranasal insulin. The study by Calkin CV et al. suggests that reversal of IR by metformin offers a therapy for treatment-resistant bipolar disorder (TRBD) [67]. A recent meta-analysis does not support the efficacy of antidiabetics being superior to the placebo in the treatment of unipolar and bipolar depression [132]. A pilot study showed metformin treatment may improve IR in schizophrenia subjects [133].

**Table 5:** Treatments of insulin resistance.

Treatment	Outcome	Study
Intranasal Insulin	Intranasal administration can bypass the BBB and reach the CNS within 1 h. It may be able to improve synaptic plasticity and regional glucose update. This may serve to alleviate the neuropathology seen in AD. It has also been noted to improve memory performance in both AD patients and healthy individuals.	Born et al. (2002) [134]
	In both MCI and AD patients, when treated for 12 months, there was a reduced progression of WMH [95% CI] = 18.98 [−1.38, 39.33].	Kellar et al. (2021) [130]
	It was found that patients with AD or MCI treated with intranasal insulin showed improved global cognition (SMD = 0.22, 95% CI: 0.05–0.38 *p* =< 0.00001, *n* = 12 studies)	Wu et al. (2023) [135]
Oral Antidiabetics	Oral antidiabetics, such as pioglitazone and liraglutide, were found to be effective on depressive and cognitive symptoms.	Possidente et al. (2023) [136]
Metformin	Cognitive function improved in patients with schizophrenia when treated with metformin and antipsychotics compared to those with just antipsychotics. It was thought to be associated with enhanced functional connectivity of the DLPFC. Significantly greater improvements in the areas of speed of processing, working memory, verbal learning, and visual learning were seen in patients receiving metformin	Shao et al. (2023) [137]
	When exploring the effects of metformin on cognitive impairment, a study looking at 365 individuals (≥55 years old) found that long term use of metformin was associated with a decrease in cognitive decline (OR: 0.49 [CI 0.25–0.95]).	Ng et al. (2014) [138]
	When examining the protective effect of metformin on cognitive decline and the development of AD, it was found that metformin use was not associated with incident AD (aOR 0.99; 95%, CI = 0.94–1.05). The authors also noted that long-term use at high doses was associated with a lower risk of incident AD in older individuals with T2DM.	Sluggett et al. (2020) [139]
Incretins/GLP1 Receptor Agonists	The REWIND study examined the effect of once-weekly subcutaneous injection of dulaglutide on cognitive impairment. When compared to the placebo group, the hazard of significant cognitive impairment in those receiving the dulaglutide was reduced by 14% (HR 0.86 95% CI: 0.79–0.95 *p* = 0.0018).	Cukierman-Yaffe et al. (2020) [140]
DPP4 Inhibitors	In individuals with T2DM and MCI, those that received a DPP4 inhibitor were protected against worsening cognitive impairment.	Rizzo et al. (2014) [141]
	An RCT of individuals with T2DM received sitagliptin for 6 months, and there was an associated increase in MMSE scores (*p* = 0.034). In patients with AD that received sitagliptin, there was an improvement in cognitive function compared to those receiving metformin (*p* = 0.047).	Isik et al. (2017) [142]
P-par gamma Agonists	When individuals with mild-to-moderate AD were given rosiglitazone or donepezil (control). For those receiving donepezil, no significant treatment difference was detected in ADAS-Cog; however, a significant difference was detected (*p* = 0.009) on the CIBIC+. The authors found no evidence of efficacy of rosiglitazone monotherapy in cognition or global function.	Gold et al. (2010) [123]
	Individuals with mild-to-moderate AD were treated with pioglitazone or other oral hypoglycemic medications (control). At month 6, the ADAS-cog scores decreased significantly in the pioglitazone group (*p* < 0.05), while they increased significantly in the control group (*p* < 0.05). The WMS-R logical memory-I scores significantly increased in the pioglitazone group (*p* < 0.01) but not in the control group.	Hanyu et al. (2009) [143]
SGLT Inhibitors	The rate of incident AD was assessed in individuals taking an SGLT2 inhibitor versus a DPP-4 inhibitor. When comparing SGLT2 inhibitors with DPP-4 inhibitors, SGLT2 inhibitors had a lower risk of dementia (14.2/1000 person-years; aHR 0.80 [95% CI 0.71–0.89]). Dapagliflozin exhibited the lowest risk (aHR 0.67 [95% CI 0.53–0.84]), followed by empagliflozin (aHR 0.78 [95% CI 0.69–0.89]), whereas canagliflozin showed no association (aHR 0.96 [95% CI 0.80–1.16]).	Wu et al. (2023) [144]

Alzheimer dementia—AD, Alzheimer’s Disease Assessment Scale-cognitive subscale—ADAS-Cog, Alzheimer’s Disease Assessment Scale-Cognitive subscale (Japanese version)—ADAS-Jcog, blood–brain barrier—BBB, central nervous system—CNS, Clinician’s Interview-Based Impression of Change plus caregiver input—CIBIC+, confidence interval—CI, Dipeptidyl peptidase-4—DPP4, dorsolateral prefrontal cortex—DLPFC, glucagon-like peptide 1—GLP1, hazard ratio—HR, mild cognitive impairment—MCI, mini-mental status examination—MMSE, peroxisome-proliferator-activated receptor—P-par, randomized control trial—RCT, sodium-dependent glucose cotransporter—SGLT, Type 2 diabetes mellitus—T2DM, Wechsler Memory Scale-Revised logical memory-I—WMS-R, White matter hyperintensity—WMH.

## 4. Conclusions

Insulin resistance plays an important role in the causation of AD and other neuropsychiatric diseases through pathways like impaired insulin signaling, inflammation, and disrupted glucose metabolism. IR may be one of the key drivers for cognitive and psychiatric disorders, and addressing it may help to improve the management of these conditions. Developing cost-effective and reliable biomarkers for early identification and management of neuropsychiatric disorders is essential. This review showed therapeutic approaches like medications, exercise, intermittent fasting to brain disorders centered around insulin signaling and metabolism, as well as insulin resistance. It looks like the cornerstone for the treatment of IR at this point includes weight loss and exercise. Research from weight loss and exercise shows a promising simple lifestyle management of these conditions. Preliminary research has indicated that these treatments have beneficial effects on neuropsychiatric disorders, but comprehensive and long-term research is necessary to fully assess their efficacy. To apply this evidence in clinical practice, more research is necessary.

## Figures and Tables

**Table 1:** Role of insulin receptors in different cells of the brain—adapted and modified from Arnold S, 2018 [9].

Cell Type	Role of Insulin Receptor
Neuron	The predominant isoform is IRα. In both the presynaptic and postsynaptic compartments are the IR, IRS1, and IRS2. Ion channel regulation and localization for GABA, NMDA, and AMPA receptors. Catecholamine release modulation. LTP and LTD balance regulation. Neurogenesis promotion and apoptosis inhibition. Trafficking of GLUT3 and GLUT4.
Microglia	IR, IRS1, and IRS2 are present. Modulation of cytokine production and the inflammatory response.
Astrocytes	The main form is IRβ. Signaling via IRS1 and IRS2. Promotes glycogen storage and encourages the uptake of glucose across the BBB. Helps to modulate inflammatory cytokine secretion.
Oligodendrocytes	There is the presence of IR, IRS1, and IRS2. Role of insulin and its effects are not fully understood.
Arterioles, Capillaries, and the BBB	Insulin transport into the brain across the BBB is IR-mediated. Helps to regulate the BBB GLUT1 expression. Involved in the promotion of NO-mediated vasodilation allowing for the increased cerebral perfusion.

Alpha—α; α-Amino-3-hydroxy-5-methyl-4-isoxazolepropionic acid—AMPA; Beta—β; blood–brain barrier—BBB; gamma-aminobutyric acid—GABA; glucose transporter—GLU; insulin receptor—IR; insulin receptor substrate—IRS; long-term depression—LTD; long-term potentiation—LTP; N-methyl-D-aspartate—NMDA; nitrogen monoxide—NO.

**Table 2:** Action of insulin signaling in the brain—adapted and modified from Chen W et al. 2022 [12].

Brain Cognition	Cells Involved	Proposed Mechanism of Action	Outcome
Normal Conditions	Astrocyte	Increased ATP and dopamine release	Impact on the reward circuitry
	Neuron	Involvement of GABA-R and Glutamate-R neuropeptides Involvement of p-NMDAR and p-AMPAR and cholesterol synthesis	This supports synaptic plasticity and synaptogenesis, which can impact cognition.
	Tanycyte	Interaction with the hypothalamic circuit	It can be involved with synaptic plasticity and synaptogenesis, involved with cognition. It can also have an impact on appetite control and systemic metabolism.
Insulin resistance (T1DM, T2DM, Obesity)	Astrocytes	Decreased p-Munc18c activation, leading to decreased ATP release and potentially increased dopamine release	Influence on mood and potential disorders
	Degenerating Neurons	This leads to increased mitochondrial dysfunction, leading to increased inflammation and oxidative stress. This can enhance neuronal apoptosis.	This can lead to impairments in cognition and potential AD.
	Activated Microglia	Increased activity of p-GSK3β, leading to a tau-phosphorylation increase, which can lead to abnormal neural activities and networks.	This can also impact cognition and lead to potential AD.

α-amino-3-hydroxy-5-methyl-4-isoxazolepropionic acid—AMPA; adenosine triphosphate—ATP; Alzheimer Dementia—AD; γ-aminobutyric acid—GABA; glycogen synthase kinase-3 beta— GSK3β; mammalian uncoordinated 18c—Munc18c; Type 1 diabetes mellitus—T1DM; Type 2 diabetes mellitus—T2DM.

**Table 4:** Peripheral and central features of insulin resistance.

Insulin Resistance	Outcome	Reference
Peripheral	Hyperglycemia Hypertension Microalbuminuria Low High-density lipoprotein Increased triglycerides Dark dry, velvety skin in arm pits, groin, back of neck (Acanthosis Nigricans) Central (visceral obesity) Increased fibrinogen Increased plasminogen activator Increased uric acid levels	Kraemer et al. (2014) [19]
Central	Inadequate central control over the distribution of nutrients Cognitive and mood problems Brain-specific neuropathology Neurodegeneration.	Arnold et al. (2018) [9]

## Data Availability

No new data were created or analyzed in this study. Data sharing is not applicable to this article
